# How Does ADPKD Severity Differ Between Family Members?

**DOI:** 10.1016/j.ekir.2024.01.053

**Published:** 2024-02-05

**Authors:** Klement C. Yeung, Elise Fryml, Matthew B. Lanktree

**Affiliations:** 1 Temerty Faculty of Medicine, University of Toronto, Toronto, Ontario, Canada; 2 Department of Medicine, University of British Columbia, Vancouver, British Columbia, Canada; 3 Department of Medicine, McMaster University, Hamilton, Ontario, Canada; 4 Division of Nephrology, St. Joseph’s Healthcare Hamilton, Hamilton, Ontario, Canada; 5 Department of Health Research Methodology, Evidence, and Impact, McMaster University, Hamilton, Ontario, Canada; 6 Population Health Research Institute, Hamilton, Ontario, Canada

**Keywords:** ADPKD, genetics, incomplete penetrance, intrafamilial discordance, polycystic kidney disease, variable expressivity

## Abstract

Thousands of pathogenic variants in more than 100 genes can cause kidney cysts with substantial variability in phenotype and risk of subsequent kidney failure. Despite an established genotype-phenotype correlation in cystic kidney diseases, incomplete penetrance and variable disease expressivity are present as is the case in all monogenic diseases. In family members with autosomal dominant polycystic kidney disease (ADPKD), the same causal variant is responsible in all affected family members; however, there can still be striking discordance in phenotype severity. This narrative review explores contributors to within-family discordance in ADPKD severity. Cases of biallelic and digenic inheritance, where 2 rare pathogenic variants in cystogenic genes are coexistent in one family, account for a small proportion of within-family discordance. Genetic background, including cis and trans factors and the polygenic propensity for comorbid disease, also plays a role but has not yet been exhaustively quantified. Environmental exposures, including diet; smoking; alcohol, salt, and protein intake, and comorbid diseases, including obesity, diabetes, hypertension, kidney stones, dyslipidemia, and additional coexistent kidney diseases all contribute to ADPKD phenotypic variability among family members. Given that many of the factors contributing to phenotype variability are preventable, modifiable, or treatable, health care providers and patients need to be aware of these factors and address them in the treatment of ADPKD.

ADPKD affects about 1 in 1000 people, and approximately 85% of those affected have a known family history.^1^^,^^2^ Although a new or *de novo* mutation accounts for as many as a quarter with no family history,^2^ a positive family history of ADPKD can also be missed due to familial estrangement, death in family members due to other causes before a diagnosis of ADPKD is identified, or lack of access to medical care in family members. Thus, clinicians should delve into the difference between a known negative family history and an unknown family history. Treating patients with ADPKD without a positive family history can come with different challenges, because the diagnosis is a surprise and completely new to the patient. In contrast, patients with exposure to the course of ADPKD in an affected parent often worry their disease course will mirror their parents. Examination of factors contributing to within-family discordance or intrafamilial discordance in phenotype severity can provide insights into ADPKD pathogenesis and empower patients to optimize their long-term health regardless of the presence of family history.

Development of bilateral kidney cysts that grow throughout life leading to an increase in total kidney volume (TKV) is pathognomonic of ADPKD. Additional kidney manifestations include flank pain, kidney stones, cyst infections, and hypertension. Cysts outside of the kidney are also common in ADPKD, including hepatic and pancreatic cysts. Mitral valve prolapse, aortic insufficiency, and intracranial and arterial aneurysms are also noted to be more prevalent in patients with ADPKD.^3^ In published cohorts of patients with ADPKD, the age at development of kidney failure ranges from pediatric-onset to avoiding it altogether, but the mean age is between fifth and seventh decade.^4^^,^^5^ By ADPKD “phenotype severity” we are referring to adverse kidney outcomes, including the rate of growth in TKV, the loss of kidney function by estimated glomerular filtration rate (eGFR), and the age at development of kidney failure. Examination of within-family variation of extra-renal ADPKD manifestations such as cystic liver disease or intracranial aneurysms is beyond the scope of the current review.

Ascertainment of patients with classic severe ADPKD phenotypes can yield *PKD1* variants in up to 85% of solved patients.^6^, ^7^, ^8^, ^9^ However, in general nephrology clinics where patients are referred with multiple kidney cysts but normal or near-normal kidney function, as many as one-third will have no *PKD1* variant and 15% have no pathogenic variant identified.^4^ In the 85% of cases where pathogenic variants are found, ADPKD is the result of a rare pathogenic variant in *PKD1* in 70% of cases and *PKD2* in 30% of cases.^10^ This ratio of observed *PKD1:PKD2* cases closely resembles the ratio of *PKD1:PKD2* variants seen in population sequencing data and is in keeping with the size of the respective *PKD1* and *PKD2* genes, and thus the likelihood of rare variants occurring in them by chance.^1^ Variants can be further subclassified into truncating (nonsense, frameshift, or splicing) or nontruncating (missense or in-frame insertions or deletions). Multiple cohorts around the world have now demonstrated a genotype-phenotype correlation with the most severe disease associated with *PKD1* truncating variants, followed by *PKD1* nontruncating variants and the mildest disease associated with *PKD2* variants.^11^, ^12^, ^13^ Nonetheless, substantial variability in kidney disease severity exists for a given mutation class and individuals with *PKD1* truncating mutations can still have relatively mild disease.^5^ The Mayo Clinic ADPKD imaging classification is the best tool for risk stratification of both the future rate of loss of kidney function and the risk of patient-important outcomes, because it incorporates the impact of both genetic and environmental factors on disease severity up to that point in life.^14^^,^^15^ Notably, about 10% of patients with atypical or “class 2” cyst distributions, described as unilateral, segmental, asymmetric, lopsided, or with atrophy, were excluded from the Mayo Clinic classification, but have a mild prognosis and are less likely to have a pathogenic *PKD1* or *PKD2* variant.^16^

Exome sequencing of patients with clinically suspected ADPKD but no pathogenic *PKD1* or *PKD2* variant identified, which can account for up to as many as 15% of patients with multiple kidney cysts, has now identified more than 13 cystogenic genes (i.e., *ALG8*, *ALG9*, *DNAJB11*, *GANAB*, *HNF1B*, *IFT140*, *SEC61B*, *PKHD1*, *PRKCSH*, *SEC63*, *COL4A3/4/5*) that account for up to half of these initially genetically unresolved ADPKD cases.^17^ Pathogenic variants in *DNAJB11* are associated with cyst formation without kidney enlargement, but in contrast to ADPKD due to *PKD1* or *PKD2* mutations, also demonstrate chronic interstitial fibrosis.^8^ Autosomal dominant polycystic liver disease, which can also have kidney cyst manifestations are associated with pathogenic variants in *ALG5*, *ALG8*, *ALG9*, *SEC61B*, *SEC63*, *GANAB*, and *PRKCSH*.^18^, ^19^, ^20^, ^21^, ^22^ The kidney phenotype in these patients with non-*PKD1* non-*PKD2* ADPKD phenotypes are generally less severe and kidney failure is not usually observed without an additional contributor to kidney disease severity.^18^^,^^20^ In patients where no rare pathogenic variant is identified, the phenotype is also typically milder than in those with ADPKD due to *PKD1* or *PKD2* mutations.^2^ In total, variants in over 100 different genes have been reported to cause kidney cysts, but usually with substantially different or additional phenotypes (See Clinical Genome Resource Kidney Cystic and Ciliopathy Disorders Expert panel: https://clinicalgenome.org/affiliation/40066/).

In studies of twins with ADPKD from the early 2000s, monozygotic twins reached kidney failure within 2 years of each other on average.^23^ Persu *et al.* also reported that the difference in age at kidney failure between siblings with ADPKD was 7 years on average.^23^ More recently, we identified that the larger the family, the more likely you were to observe 2 members with discordant ages at kidney failure, which we defined as greater than 15 years difference in age at kidney failure.^24^ This 15-year difference was present in 30% of families with more than 5 affected members.^24^ Conversely, we found that the age at development of kidney failure among family members was within 8 years in two-thirds of families (Figure 1). Interestingly, the rate of intrafamilial discordance was similar regardless of the type of main effect pathogenic variant (i.e. *PKD1* or *PKD2,* truncating or nontruncating) that caused the ADPKD in the family.^24^

**Figure 1:**
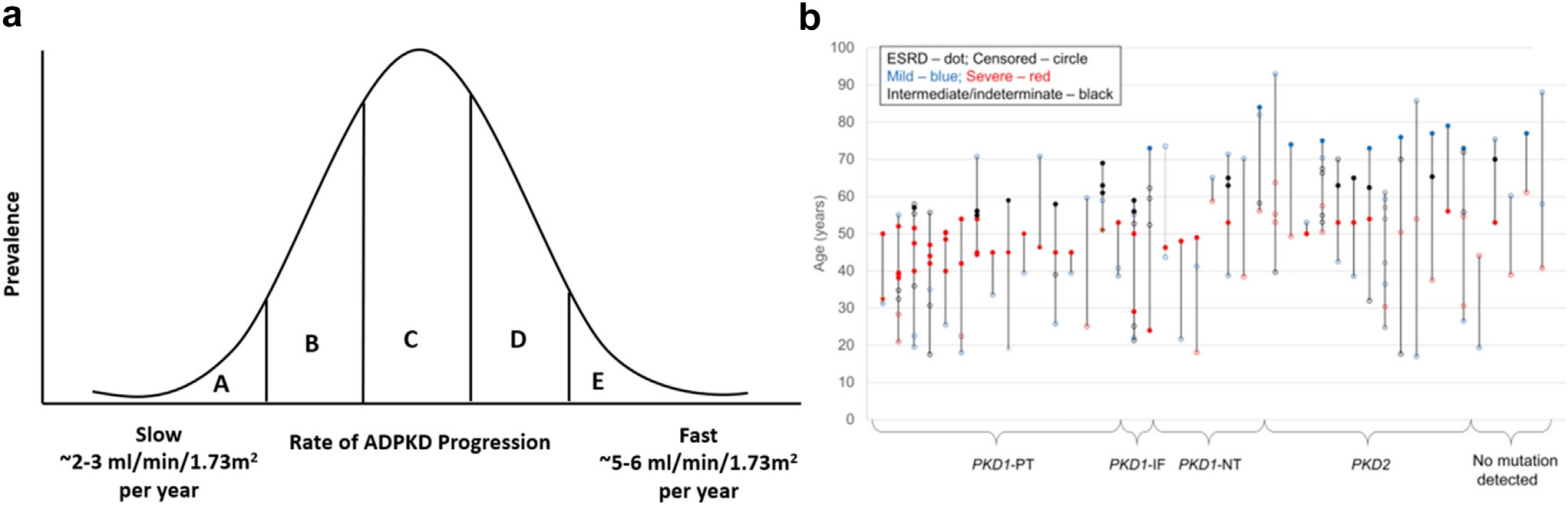
Variability in ADPKD severity within family members. (a) The rate of ADPKD progression and eGFR decline is fairly linear throughout life in any one individual and falls on a distribution compared to all others with ADPKD, ranging from slow (i.e., less than 3 ml/min per 1.73 m^2^ per year) to fast (i.e., greater than 5 ml/min per 1.73m^2^ per year). The rates of ADPKD progression correspond with the Mayo clinic imaging classification severity: A (mild) to E (severe). (b) Depiction of intrafamilial variability in disease severity. Each vertical line represents a family, ordered by the main effect pathogenic variant type. Filled dots represent age at kidney failure, while open dots represent age at censor. Families with intrafamilial discordance are those containing a blue and red dot (reproduced from Lanktree^25^). ADPKD, autosomal dominant polycystic kidney disease; eGFR, estimated glomerular filtration rate; IF, inframe deletion or duplication; NT, nontruncating; PT, protein truncating.

A question thus arises—if ADPKD is caused by a single causal pathogenic variant, and family members carry the same variant, why do some family members have different disease severity? In this review, we discuss potential contributors to intrafamilial discordance in ADPKD phenotype severity.

### Methods

To ensure a comprehensive rapid review, we performed a search for relevant peer-reviewed research articles published from January 2012 to March 2023 in English on Pubmed. The following keywords were used: “ADPKD” or “polycystic kidney”, and “risk” or “progression” (Supplementary Methods). The search yielded 872 articles after removing duplicates. Based on review of the titles and abstracts by 2 independent reviewers, a total of 100 English articles were identified to be relevant after the title and abstract screening and underwent full-text review. Additional articles were added based on reference listing and previous knowledge of the authors. These articles were reviewed, those of similar content areas were grouped and the information was incorporated in developing different sections of this review article.

### Genetic Factors Influencing Phenotype Severity

#### Haploinsufficiency and “Two-hit Models”

There are 2 schools of thought regarding the pathophysiology of cystogenesis in ADPKD, though they are not mutually exclusive. The “haploinsufficiency model” proposes that cysts develop when the level of functional polycystin complex in the cilia falls below a cystogenic threshold. In the second model, the “two-hit hypothesis”, biallelic inactivation of both copies of the *PKD1* or *PKD2* genes via somatic mutation is required for cyst formation, making the disease effectively recessive at the cellular level.^26^ In addition to the germline pathogenic *PKD1* or *PKD2* variant, acquired somatic mutations in the other copy of the gene has been identified in DNA extracted from clonally expanded cyst cell populations.^27^ However, the expectation that every single cyst requires a *de novo* somatic mutation event seems inconsistent with the rate of somatic mutation development.^28^ Hypomorphic variants, which reduce the quality of functional polycystin protein but do not result in nonsense-mediated mRNA transcript decay or an absence of the protein product, are also consistent with the cystogenic threshold model.^29^ It appears likely that both “haploinsufficiency” and “two hit” models contribute to cyst formation and growth.

In addition to somatic mutations leading to the inactivation of the second wild*-*type copy of the *PKD1* or *PKD2* gene, in those without a pathogenic variant identified, a *de novo* mutation can occur after fertilization during development resulting in mosaicism and variable dosage of the mutated allele throughout the body. Atypical asymmetric cyst patterns on imaging have been reported as a consequence of *de novo* somatic variants.^2^ If the acquired *de novo* variant impacts germ cells, the subsequent generation may be more severely affected than the parent, because all cells carry the pathogenic variant in the offspring. Systematic evaluation of an ADPKD cohort using next-generation sequencing which is more sensitive in identifying somatic variants than traditional Sanger sequencing indicates somatic variation is present in less than 1% of ADPKD cases.^30^

#### Cis and Trans PKD1 or PKD2 Variants

Two variants residing on the same chromosome, and thus inherited from one parent, are “in cis.” Contrarily, 2 variants on opposite chromosomes, 1 inherited from each parent, are “in trans.” Biallelic pathogenic *PKD1* or *PKD2* variants (i.e., trans variants with 1 inherited from both parents) lead to a more severe phenotype of polycystic kidney disease.^31^, ^32^, ^33^, ^34^ Two variants in cis are in linkage disequilibrium on the same chromosome and thus inherited together. Cis-regulatory elements are stretches of noncoding DNA that impact the expression of nearby genes on the same chromosome. MicroRNA (miR) are short (about 22 bases long) noncoding stretches of transcribed RNA produced elsewhere in the genome, that can bind to complementary stretches of mRNA awaiting to be translated that act as posttranscriptional regulators of protein production. One miR in particular, *miR-17*, regulates numerous genes in cystogenic pathways, including *PKD1* and *PKD2,* as well as *PKHD1* and *HNF1B.* Deleting the miR-17 binding motif in the 3’ untranslated region of the *Pkd1* gene reduces cis-inhibition, thereby increasing—or so-called “derepressing”—the amount of polycystin from the functioning wild-type copy of the gene, and thus reducing cyst severity in mice models (Figure 2).^35^ These findings raise the possibility that differences within cis-regulatory elements of the functioning wild-type copy of *PKD1 or PKD2* on the opposite chromosome as the pathogenic variant*,* which would be expected to vary between affected family members, could lead to variability in the development and progression of ADPKD between family members. It should be noted that this phenomenon has not yet been reported in humans but inhibiting miR-17 with another oligonucleotide (RGLS8429) is a therapeutic strategy currently under investigation.^35^^,^^36^

**Figure 2:**
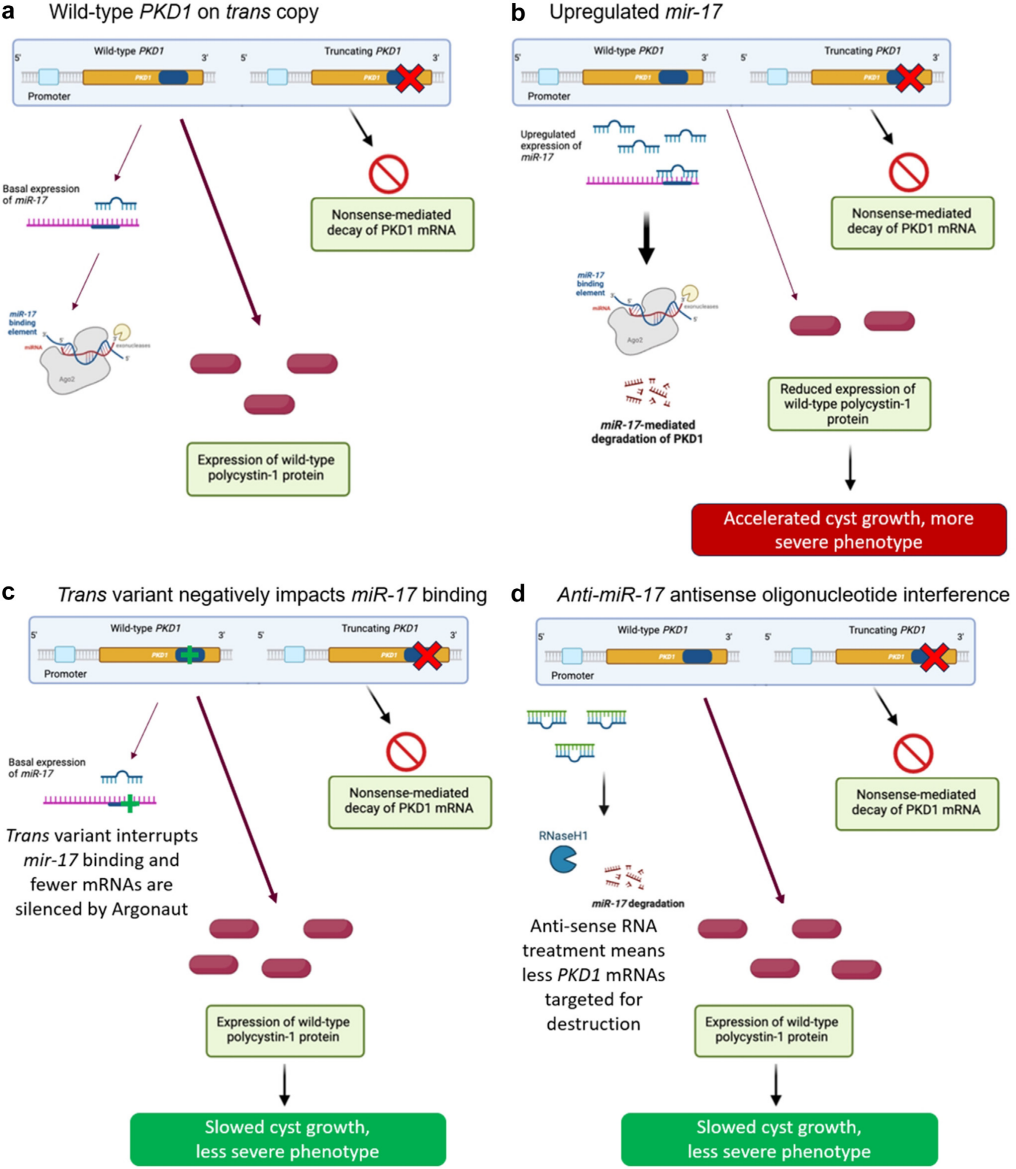
Trans genetic factors impact the expression of the wild-type copy of PKD1 in mice which could contribute to variable phenotypes among family members. MicroRNA (miR) 17 is trans acting factor that marks *PKD1* transcripts for translational repression via Argonaut (a). Upregulated miR-17 expression exacerbates the ADPKD phenotype by reducing the expression of the wild-type *PKD1* (b), whereas inhibition of miR-17 via a genetic variant in the wild*-*type copy of *PKD1* (c) or interference of mir-17 using antisense oligonucleotides now being tested in humans (d) results in increased polycystin-1, slowed cyst growth, and reduced disease severity in ADPKD models. ADPKD, autosomal dominant polycystic kidney disease; miR, microRNA.

#### Digenic Inheritance and Modifier Genes

Digenic inheritance refers to when a heterozygous pathogenic variant lies within 2 separate genes that could include *PKD1* and *PKD2* or potentially in any of the additional cystogenic genes such as *HNF-1ꞵ* or *PKHD1*.^31^ Although these rare variant modifiers only account for a small percentage of within-family variability, they can at times be identified in individuals with severe phenotypes and early age of disease onset. The role of cotransmission of additional forms of genetic kidney disease, whether associated with cystic kidney disease or not, including type IV collagen nephropathy, remains relatively unexplored.^37^^,^^38^

#### Polygenic Inheritance

Going beyond the specific genes that cause monogenic ADPKD to variants across the genome, there are known genetic risks for obesity, dyslipidemia, hypertension, diabetes, and chronic kidney disease (CKD) in general from across the genome, which could in turn contribute to the rate of kidney function decline in ADPKD.^39^ The variants that lead to this polygenic risk are typically common and with individually small effects but can be added together into polygenic scores with larger effects (odds ratios typically ∼2–4 for those within the top 5 percentile scores).^39^ Because of Mendel’s law of independent assortment, the risks of inheriting high polygenic CKD or metabolic risk are independent of monogenic causes of ADPKD. Siblings share on average 50% of their genome; thus, siblings’ polygenic risks would be correlated across a population; however, any pair of siblings can range from no shared genetic background (alternate chromosomes were allocated for every chromosome during meiosis) to the same polygenic risk (identical twins). The cumulative impact of rare variants and common variant polygenic risk can be referred to as the “omnigenic risk” (Figure 3).

**Figure 3:**
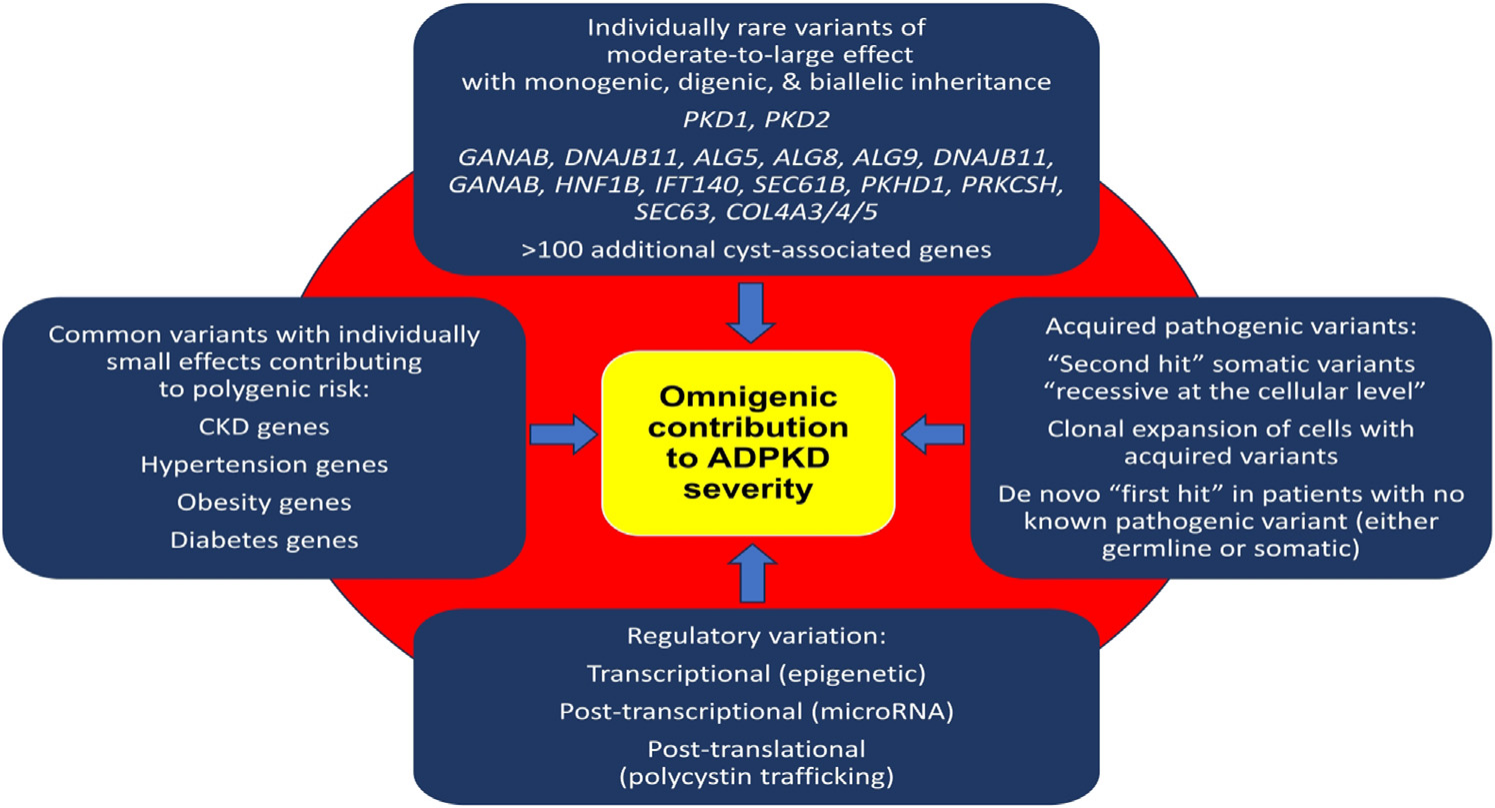
Rare variants, common variants, acquired variants, and regulatory variations cumulatively impact the omnigenetic contribution to ADPKD severity. ADPKD, autosomal dominant polycystic kidney disease; CKD, chronic kidney disease.

#### Epigenetics

Epigenetics involves the packaging of our DNA. Histones are proteins the DNA is wrapped around, and the acetylation, methylation, and crotonylation of amino acids and nucleotides impact how easily a gene can be transcribed to produce a new mRNA transcript.^40^^,^^41^ Moreover, although epigenetic signatures are inherited, they can also change throughout life in response to exposures. Further study of the impact of epigenetics on ADPKD is required.

#### Genetic Ancestry

Whether ancestry impacts the prevalence or rate of ADPKD progression has been understudied and requires further attention.^25^ In a 2021 cross-sectional analysis of 3868 patients with ADPKD within the Californian Kaiser Permanente health system, ADPKD appeared more common in non-Hispanic White (63.2 per 100,000) and Black patients (73.0 per 100,000) compared with Hispanic (39.9 per 100,000) and Asian (48.9 per 100,000) patients.^42^ In population sequencing data, there was no convincing evidence of variation in ADPKD pathogenic variant prevalence across South Asian, European, Finnish, East Asian, Latino, or African populations.^1^

#### Genetic Sex

For unclear reasons, males have worse ADPKD severity than females. Among French *PKD1* variant carriers, the median age for kidney failure was 56 years in males and 60 years in females.^11^ In the Mayo clinic and Consortium for Radiologic Imaging Studies of Polycystic Kidney Disease cohorts, males reached kidney failure on average 5.7 years earlier.^15^ An association between maternally transmitted risk alleles and ADPKD severity was proposed but appears unlikely, given that it is an autosomal condition. Choukroun *et al.* studied 79 patients with ADPKD and reported that male patients in whom the pathogenic variant was maternally inherited had an earlier age of kidney failure (46 vs. 54 years; *P* < 0.01).^43^ In contrast, in a separate report of 119 families with a *PKD1* variant, there was no difference in the age at development of kidney failure between those with maternally inherited compared to paternally inherited risk allele.^44^ In a cohort of 296 patients with information from 39 families, the age at kidney failure was in the opposite direction with worse severity when the risk allele was paternally inherited (52 vs. 61 years, *P* < 0.001).^10^ Therefore, male sex is associated with a more severe ADPKD phenotype but whether it matters if the risk allele is paternally or maternally inherited remains unlikely.^44^, ^45^, ^46^

### Environmental Factors Influencing Phenotype Severity

#### Obesity, Caloric Intake, and Fasting

In a *post hoc* analysis of 441 Halt Progression of Polycystic Kidney Disease participants, being overweight (body mass index of 25–29.9) or obese (body mass index >30) was associated with a faster rate of TKV growth and more rapid decline in eGFR.^47^ Overweight groups were also shown to have a higher rate of radicular (odds ratio = 2.31) and back pain (odds ratio = 1.88).^48^ Given the association between increased body mass index and ADPKD phenotype severity, dietary measures such as caloric restriction and intermittent fasting could contribute to improved ADPKD severity. Moreover, metabolic reprogramming and the Warburg effect, the preferential use of oxidative glycolysis as an energy source, and the upregulation of AMP-activated protein kinase signaling in ADPKD give a rationale for reduced caloric intake as a therapy.^49^ In rodent models, restriction of food intake slowed ADPKD progression, reduced cystogenesis, and improved molecular markers of inflammation, kidney injury, and fibrosis.^50^^,^^51^ Time-restricted feeding without caloric restriction also suppressed cyst proliferation and kidney fibrosis in a rat model.^52^ Moreover, a ketogenic diet showed similar benefits in a rodent model suggesting it could be attempted as a treatment.^52^ Translating findings from rodent models to humans, Hopp *et al.* randomized 28 adults with ADPKD to daily caloric restriction or intermittent fasting for 12 months. Both groups had weight loss which may have had a beneficial impact on TKV growth, supporting the feasibility of a larger study.^53^ Chebib *et al.* recently provided a comprehensive review on this topic, concluding that further research is required to establish the benefits and safety of these interventions.^54^ For now, extrapolating from the available data, and considering the deleterious effects of obesity on cardiovascular disease over and above ADPKD progression, a recommendation of daily caloric restriction is an important part of the nonpharmacologic management of ADPKD.

#### Salt and Protein Intake

Data from the Consortium for Radiologic Imaging Studies of Polycystic Kidney Disease and Halt Progression of Polycystic Kidney Disease suggests an association between greater urinary sodium excretion and higher TKV and greater risk of kidney failure.^55^^,^^56^ In the Developing Interventions in ADPKD study, salt and protein intake were estimated from 24-hour urine samples and multivariate analysis indicated that higher salt intake was associated with faster eGFR decline, whereas protein intake was not.^57^ Limiting salt and protein intake in line with recommendations for those with CKD is reasonable, but the evidence for limiting protein intake specifically in ADPKD is poor.

#### Water Intake

Given the mechanism of action of vasopressin V2 receptor antagonists for slowing ADPKD, reducing endogenous vasopressin release through hydration seems reasonable. However, the best available randomized evidence is inconclusive at best. Rangan *et al.* randomized 276 participants to a prescribed water intake to reduce urine osmolality to 270 mOsm/kg or less compared to *ad libitum* water intake.^58^ The intervention group achieved a mean 0.6 L increase in urine volume and a mean 91 mOsm/kg reduction in urine osmolality, but there was no difference in TKV or eGFR decline with the intervention over 3 years.^58^ Due to urinary concentrating deficits in ADPKD, many patients report high water intake at baseline. Reducing stone risk with hydration could be beneficial. However, adequately increasing water intake to meaningfully reduce vasopressin may not be feasible and should not be viewed as an alternative to pharmacologic inhibition of vasopressin in high-risk patients.

#### Cigarette Smoking

Smoking is known to be a major risk factor for vascular disease, and cardiovascular disease is the leading cause of death in patients with ADPKD.^59^ Further, patients with ADPKD who currently smoke had a 3-fold higher risk of cerebrovascular events than never-smokers.^60^ Most patients are aware that smoking is not good for them, but patients with ADPKD should be specifically counseled regarding the risks and provided cessation strategies.

#### Alcohol Intake

Alcohol ingestion inhibits the central production of vasopressin, increasing urine production and inducing dehydration. Plasma vasopressin levels are inversely correlated with alcohol intake.^61^ Inhibiting the action of vasopressin is the therapeutic target of tolvaptan, and thus a beneficial impact of alcohol on ADPKD could be hypothesized. However, given the health consequences of alcohol consumption, the impact of alcohol on ADPKD progression has not been studied. Limiting alcohol intake reduces blood pressure and malignancy risk among the general population.^62^^,^^63^ In patients with ADPKD, we recommend limiting or avoiding the use of alcohol in line with guidance for the general public.

### The Impact of Comorbid Diseases

#### Hypertension

The Halt Progression of Polycystic Kidney Disease study A trial randomized 558 early-stage (eGFR over 60 ml/min per 1.73 m^2^) patients with ADPKD to aggressive (95–110/60–75) versus standard (120–130/70–80) blood pressure targets over 5 years.^64^ Those in the aggressive target group had slower kidney growth, but no difference in eGFR decline.^64^ An analysis by Chen *et al.* found that patients with ADPKD who had a concomitant diagnosis of hypertension or diabetes or hypertension and diabetes had higher all-cause mortality.^65^

#### Diabetes

A retrospective cohort analysis reported that those with ADPKD and diabetes had earlier onset of hypertension, faster kidney growth, and more rapid decline in kidney function than those without diabetes.^66^ Moreover, a rationale exists that metformin could slow ADPKD progression over its antidiabetic effects,^67^ and reduce polyuria in tolvaptan-treated patients.^68^ Diabetes further increases the risk of ADPKD progression and early initiation of diabetic management in patients with ADPKD is advised. Whether sodium-glucose cotransporter-2 inhibitors could improve ADPKD outcomes in those with or without diabetes is unknown, because patients with ADPKD were specifically excluded from the clinical trials due to concerns about increasing endogenous vasopressin secretion.^69^ Pilot clinical trials for evaluating the feasibility of sodium-glucose cotransporter-2 inhibitors in ADPKD are now registered in clinicaltrials.gov.^70^

#### Kidney Stones

Kidney stones occur in up to 25% of patients with ADPKD, likely the result of both anatomic and metabolic risk factors.^71^, ^72^, ^73^ Patients with ADPKD and stones also have larger cysts and larger kidneys, and faster kidney function decline.^74^ The relationship is likely bidirectional with ADPKD causing stones, and mice models support that stones exacerbate ADPKD.^75^ Evaluation of kidney stone risk factors, including water intake, vitamin D and calcium intake, and avoiding salt and animal protein, and vitamin C supplementation is advisable in patients with ADPKD, especially after a stone event.

#### Dyslipidemia

Due to increased cardiovascular risk, patients 50 years or older with CKD who are not on dialysis are recommended to be treated with cholesterol-lowering treatment.^76^ Data do not exist to recommend lipid-lowering therapy in ADPKD specifically; however, *post hoc* analysis of the tolvaptan trials found that it could be used safely in combination with statins.^9^

#### Mental Health

Advanced CKD stage is negatively correlated with quality of life; and grief, anxiety, depression, and feelings of helplessness are common among those with ADPKD.^77^^,^^78^ Overall, additional research is required to draw concrete conclusions and identify strategies for improving the mental health of patients with ADPKD.

#### Complications of ADPKD

Urologic complications of ADPKD, including gross hematuria, cyst infections, and cyst ruptures are likely both signs of severe ADPKD and contributors to ADPKD progression.^45^^,^^64^^,^^79^ Vascular complications including cerebral aneurysm, aortic aneurysm, and aortic dissection are uncommon events that may exacerbate ADPKD progression.^80^

#### Coexisting kidney diseases

Patients with ADPKD may develop additional kidney diseases. Cases of coexistent ADPKD with membranous glomerulonephritis, focal segmental glomerulosclerosis, minimal change disease, IgA nephropathy, immune complex glomerulonephritis, and anti-neutrophil cytoplasmic autoantibody vasculitis have all been reported.^81^, ^82^, ^83^, ^84^, ^85^, ^86^ The potential for ADPKD to be modified by coexistent APOL1 nephropathy has not been evaluated.

### Treatment Adherence

European data from 1991 to 2010 showed improvement in overall survival but no improvement in the age at incidence of kidney failure in patients with ADPKD.^87^ Since then, tolvaptan has been proven as a cyst-directed therapy with evidence of slowing TKV growth and rate of eGFR decline.^88^^,^^89^ We are yet to see clinical data that treatments are delaying the average age at kidney failure in patients with ADPKD from population-based studies. Hopefully, recent developments in ADPKD management are moving the needle. Adherence to recommendations for both pharmacological and nonpharmacological interventions for ADPKD would be expected to contribute to variability in ADPKD progression between family members.

### Future Directions

The importance of cascade screening in familial kidney disease cannot be overstated because first-degree family members of individuals affected with an autosomal dominant condition have a 50% pretest probability of having the disease. Cascade screening represents seeking out first-degree and second-degree relatives of the index case for disease screening. Screening can occur via phenotype assessment in family members, often by ultrasound in ADPKD, or via genetic testing. Testing for a known familial variant is easier than sequencing the whole gene and is sometimes included at no cost by genetic testing providers. On the other hand, falling sequencing costs and a greater understanding of genetic modifiers will mean that comprehensive screening of the exome and even the whole genome of family members will become feasible. Moreover, cascade screening facilitates the collection of large pedigrees for further research into new treatments. Efforts to merge these pedigrees into multisite collaborations, including participants of diverse ancestral backgrounds, holds the potential for additional insights into ADPKD pathophysiology and should be the focus of research teams around the globe.

### Conclusion

In summary, the rate of kidney cyst growth and loss of kidney function varies between individuals with ADPKD, even among family members with the same causal pathogenic variant. Imaging has proven more successful than genetics as a stratification tool of risk for progression to kidney failure, because it incorporates the impact of both genetic risk and exposures throughout life. Many additional acquired and environmental factors contribute to the rate of ADPKD progression throughout life and, thus, influence intrafamilial differences in disease severity beyond the main pathogenic variant (Figure 4). These factors include additional genetic variability, environmental and dietary contributors, comorbid conditions, and treatment adherence. Understanding these factors and addressing them in the clinical management of ADPKD enhances the quality of patient care. Encouraging and empowering patients to manage their ADPKD with the following take-home messages at every clinic visit can enhance long-term outcomes: (i) achieve optimal weight through exercise and diet, (ii) avoid smoking, (iii) control blood pressure and cardiovascular risk factors, and (iv) adhere to ADPKD risk-based treatment recommendations. Finally, the study of affected sibling pairs and pedigrees is a valuable tool to uncover both inherited and environmental factors that contribute to variable disease expression in the context of the same pathogenic variant.

**Figure 4:**
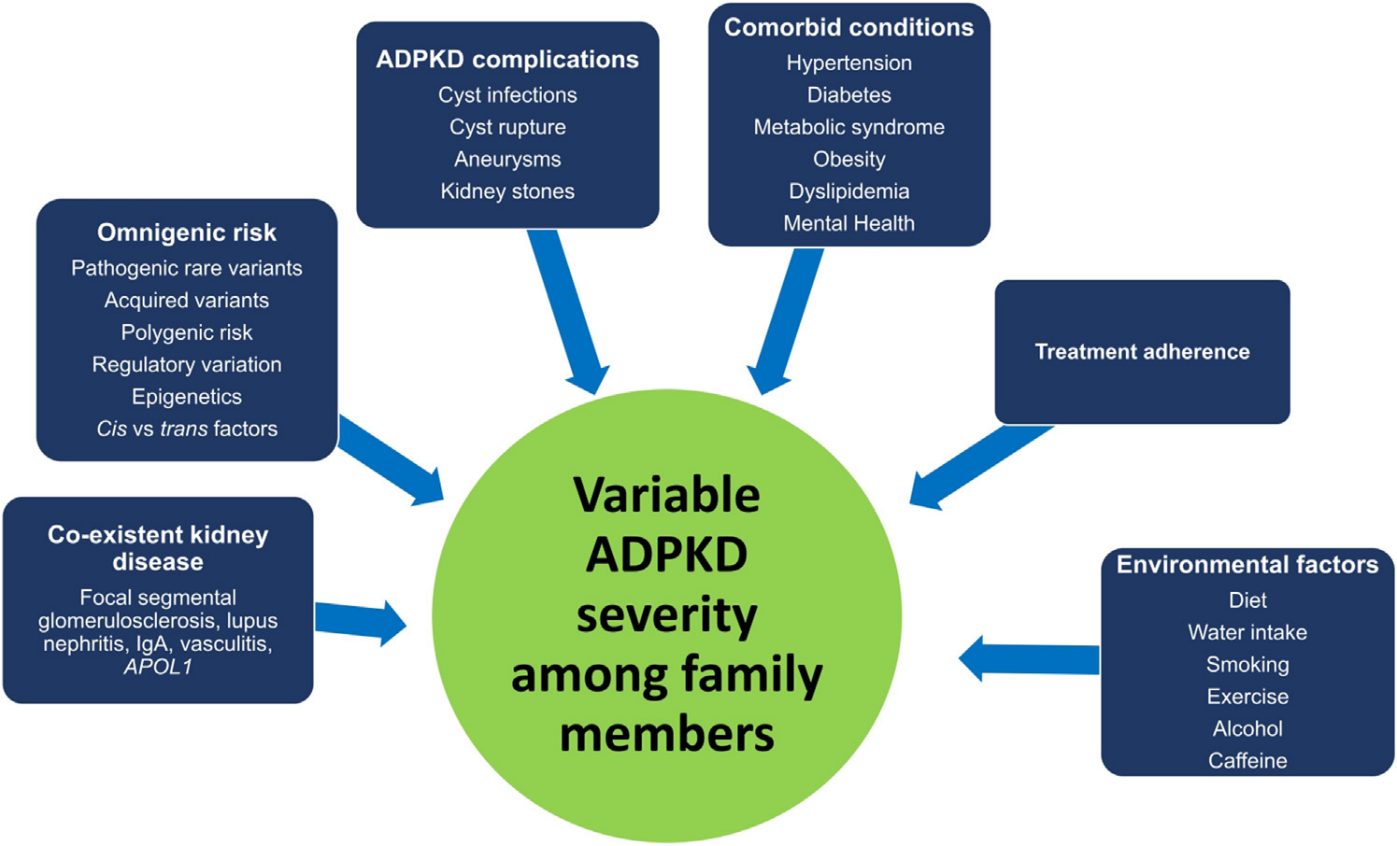
Summary of inherited, environmental, and behavioral contributors impacting variability in ADPKD severity among family members. ADPKD, autosomal dominant polycystic kidney disease.

## Disclosure

MBL has received speaker and advisory fees from Otsuka, Reata, Bayer, and Sanofi Genzyme.
