# Supplementary material for: How Does ADPKD Severity Differ Between Family Members?
Source: Kidney Int Rep. 2024 Feb 5;9(5):1198–209. doi: 10.1016/j.ekir.2024.01.053 (PMC11068977; doi:10.1016/j.ekir.2024.01.053)
Supplement: Supplementary File (PDF) [file mmc1.pdf]

Supplemental Methods:

Search Strategy

(((((ADPKD[Title]) OR (polycystic kidney[Title])) AND ((Risk) OR (Progression)) AND (("2012"[Date - Publication] : "3000"[Date - Publication]))) AND (English[Language])) AND (Human)

Database:
McMaster University Books@Ovid
Embase <1974 to 2022 August 26>
McMaster University Full Text Journals@Ovid
EBM Reviews - Cochrane Database of Systematic Reviews <2005 to August 24, 2022>
EBM Reviews - ACP Journal Club <1991 to July 2022>
EBM Reviews - Database of Abstracts of Reviews of Effects <1st Quarter 2016>
EBM Reviews - Cochrane Clinical Answers <August 2022>
EBM Reviews - Cochrane Central Register of Controlled Trials <July 2022>
EBM Reviews - Cochrane Methodology Register <3rd Quarter 2012>
EBM Reviews - Health Technology Assessment <4th Quarter 2016>
EBM Reviews - NHS Economic Evaluation Database <1st Quarter 2016>
Ovid Emcare <1995 to 2022 Week 34>
Ovid MEDLINE(R) and Epub Ahead of Print, In-Process, In-Data-Review & Other Non-Indexed Citations, Daily and Versions <1946 to August 26, 2022>

Query:
#1: polycystic kidney disease, results 27,095
#2: autosomal dominant polycystic kidney disease, results 13,147
#3: ADPKD, results 11,835
#4: 1 or 2 or 3, results 29,418
#5: genetics, results 5,219,868
#6: 4 and 5, results, 5,284
#7: limit to human, results 4,356
#8: limit to English, results 3,978
#9: limit to last 5 years, results 1,045
#10: remove duplicates, results 890
